# Friction and Wear Behavior of Polyimide Composites Reinforced by Surface-Modified Poly-p-Phenylenebenzobisoxazole (PBO) Fibers in High Ambient Temperatures

**DOI:** 10.3390/polym11111805

**Published:** 2019-11-03

**Authors:** Liang Yu, Yuanjie Zhang, Jiaming Tang, Jicheng Gao

**Affiliations:** College of Mechanical Engineering of Yangzhou University, Yangzhou 225127, China; 15861394939@163.com (Y.Z.); t779171@163.com (J.T.)

**Keywords:** friction and wear behavior, PBO fiber, surface modification, high temperature environment

## Abstract

(1) In order to improve the properties of antifriction and wear resistance of polyimide (PI) composite under high temperature conditions, (2) 3-Aminopropyltriethoxysilane (APTES) and Lanthanum (La) salt modifications were employed to manufacture poly-p-phenylenebenzobisoxazole (PBO)/PI composites with different interface properties. The representative ambient temperatures of 130 and 260 °C were chosen to study the friction and wear behavior of composites with different interface properties. (3) Results revealed that while both modification methods can improve the chemical activity of the surface of PBO fibers, the La salt modification is more effective. The friction coefficient of all composites decreases with the increase of sliding velocity and load at two temperatures, and the specific wear rate is increases. Contrary to the situation in the 130 °C environment, the wear resistance of the unmodified composite in the 260 °C environment is greatly affected by the sliding velocity and load, while the modified composites are less affected. Under the same test parameters, the PBO–La/PI composite has the lowest specific wear rate and friction coefficient, and (4) La salt modification is a more effective approach to improve the properties of antifriction and wear resistance of PI composite than APTES modification in high ambient temperatures.

## 1. Introduction

With the rapid development of high-tech industries such as energy, automobile, aeronautics, and astronautics, the development of lightweight high-strength structural materials and high-performance self-lubricating friction materials is becoming an urgent need in this field. Polyimide (PI) resin possesses the advantages of low density, high thermal stability, antifriction self-lubrication, corrosion resistance, and shock and vibration absorption, and also has the characteristics of space materials such as low volatiles, less volatile condensable materials, etc. It is a kind of potential material to be applied in high-tech friction systems such as aerospace [1,2]. Fiber reinforcement can significantly improve the mechanical and tribological properties of PI and enhance the application range. Short fiber-reinforced PI composite can be prepared by the forming process of standard hot pressure molding or vacuum autoclave for heat-resistant composite parts with complex structure and small volume. These materials not only maintain the outstanding advantages of high temperature resistance, thermo-oxidative stability, and mechanical properties at high and low temperature of polyimide composites, but also have simple processing properties, thus further expanding the application fields of high temperature-resistant resin matrix composites [3].

Poly-p-phenylenebenzobisoxazole (PBO) fiber is a rigid rod polymer composed of a benzene ring and an aromatic heterocyclic ring [4]. It presents excellent mechanical properties, antifriction and wear resistance, flame retardancy, and high temperature resistance, and its specific strength and specific modulus are in the forefront of various fibers [5,6]. PBO fiber is formed from microfibrils with 10–50 nm in diameter highly oriented to the fiber axis, and contains many capillary-like microvoids between the microfibrils [7]. The microfibrils are provided with motion space through the microvoids between them. When PBO fiber encounters dynamic load, the microvoids produce internal friction through mutual movement so as to consume large amounts of energy. Wu et al. pointed out that PBO fiber possesses better capacity in anti-vibration and energy absorption than other types of fibers such as carbon and aramid fibers (CF and AF) [8].

With PBO fiber as the reinforced phase, it is possible to obtain PI resin matrix composites with excellent tribological properties under extreme conditions. The tribological properties of PBO/PI composites depend not only on the properties of PBO fibers and PI resin matrix, but also on the efficiency of stress transfer at the fiber/matrix interface [9]. However, the characteristics of PBO fiber, chemically inert and smooth surface, prevent efficient infiltration and chemical bonding with almost all resin matrices, which severely limits the applications in the domain of advanced composite [10,11]. Therefore, the modification of the PBO fiber’s surface, which can enhance the adhesion between the PBO fiber and polymer, is of great importance to study PBO fiber reinforced resin matrix composites.

Zhang et al. dealt with the plasma-induced coating process on the surface of PBO fibers to obtain a strong interfacial adhesion between PBO fibers and the poly (phthalazinone ether sulfone ketone) (PPESK) matrix [12]. Ma et al. employed the method of hydrothermal synthesis to deposit zinc oxide (ZnO) nanoparticles on the PBO fiber surface. The results showed that the hydrophobicity and thermal stability were improved [13]. Gu et al. modified PBO fiber surfaces using silane coupling agent (KH-560) treatment assisted by ultrasonic vibration, and they found that the surface roughness of PBO fibers was increased and the single fiber pull-out strength of PBO was improved [14]. Acid treatment [15] and γ-ray irradiation [16] modification techniques were other efficient methods to enhance PBO fiber–matrix adhesion. It can be perceived that different modification methods have achieved some effects on improving certain properties of PBO fibers and their composite materials.

Lanthanum chloride ionizes La^3+^ in solution with a special 4f electronic layer structure, which has strong affinity with non-metallic elements such as N and O, and can play a “bridge” between the PBO fiber and the PI resin, improving the strength and toughness of composite interface. Lanthanum chloride is an inorganic material that is more resistant to high temperatures than traditional organosilane coupling agents, and is therefore more suitable for use in high temperature environments. Zheng et al. used lanthanum chloride to prepare rare earth solution (RES) to modify the bamboo fiber (BF), and found that the RES-modified BF bonds with the phenolic resin matrix to form a rare earth complex, thus improving the interfacial bonding properties between BF and the resin matrix [17].

At present, there is less research on the use of PBO fiber to enhance the tribological properties of resin-based composites [18,19], especially the variation of friction and wear properties under high temperature conditions. In this work, the technics of hot pressure molding formation were adopted to fabricate composite using polyimide as matrix. We adopted two surface modification methods with less mechanical damage to PBO fibers (APTES and La salt modification) to improve the surface chemical activity of PBO fibers and the thermal stability of PI composites. The representative ambient temperatures of 130 and 260 °C were chosen to study the friction and wear behavior of PBO/PI composites with different interface properties comparatively.

## 2. Materials and Methods

### 2.1. Materials

**Figure 1:**
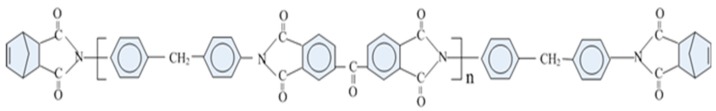
The chemical structure of polyimide resin.

The PBO fibers (Zylon, AS), supplied by Toyobo Ltd., (Kita-ku, Osaka, Japan) had an average diameter of 12.8 μm, a density of 1540 kg/m^3^, a length to diameter ratio of 234, and a thermal decomposition temperature of 700 °C. The high temperature resistant polyimide resin was supplied by Shanghai Synthetic Resin (Shanghai, China) Research Institute, and the chemical structure is shown in Figure 1.

LaCl_3_ applied as the main reagent for the La salt modification was supplied by Shanghai Yuelong New Materials Co., Ltd. (Shanghai, China), and 3-Aminopropyltriethoxysilane for APTES modification was supplied by Aldrich Chemical Company (Milwaukee, WI, USA), and other reagents used in the test were analytically pure.

### 2.2. Preparation of Modified PBO Fiber

**Figure 2:**
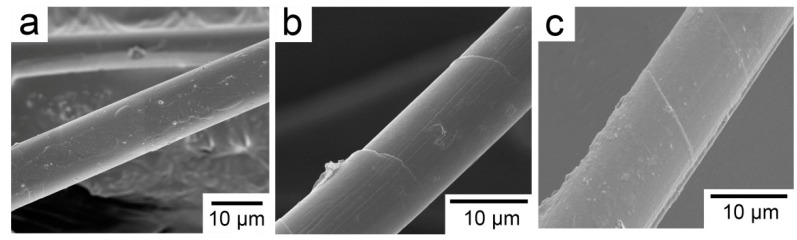
SEM photographs of (**a**) PBO-Pre fiber; (**b**) PBO-APTES fiber; (**c**) PBO-La fiber.

In order to remove the contaminants on the fiber surface and facilitate surface modification, the PBO fibers were sequentially subjected to reflux extraction with ethanol and acetone for 12 h, respectively, and then were soaked in a 60 wt % methylsulfonic acid solution at 60 °C for 1 h, washed, and dried in a vacuum oven. The resulting fiber properties were similar to those of the pristine fiber and were designated PBO-Pre fiber. La salt modifier consisted of LaCl_3_, EDTA (salt), and NH_4_Cl. The pH of the modifier was adjusted to about 6 with HCl or NaOH solution. The value of La content was 0.6 wt %. The PBO-Pre fiber mentioned above was immersed in the La salt modifier at normal temperature, soaked for 1 h, and then dried in a vacuum oven, and the obtained fiber was recorded as PBO-La fiber. For the APTES modification, the PBO-Pre fiber was immersed in a 2 wt % APTES/ethanol solution at room temperature for 1 h, and then dried in a vacuum oven to obtain PBO-APTES fiber. The SEM photographs of the fiber surface after the treatment process are shown in Figure 2. It can be seen that the surface of the PBO-Pre fiber is not damaged and is still relatively smooth. After two kinds of surface modification, the overall surface morphologies of the fibers do not change much, but only some fine grooves and protuberances are added. This indicates that the two surface modifications have less sacrifice for the mechanical properties of PBO fibers.

### 2.3. Fabrication of PBO/PI Composites

The PBO fiber and the PI resin solution were mixed at a volume ratio of 4:1 at room temperature and under nitrogen atmosphere, and the mixture was stirred uniformly. The obtained homogeneous system was vacuum dried at 80 °C for 4 h, dried at 140 °C for 2 h, dried at 200 °C for 1 h, and vacuum dried at 220 °C for 1 h, and a molding powder of a PI composite was obtained. The molding powder was placed in a metal mold at 270 °C, a contact pressure was applied, the temperature was raised to 290 °C, and the temperature was maintained for 15 min. A pressure of 2.1 MPa was applied, and the temperature was further raised to 320 °C, and the temperature was maintained for 120 min. Then, the material was cooled to room temperature to demolded and machined into the desired size.

### 2.4. Testing Procedure

The chemical state of the surface of the PBO fiber before and after modification was characterized by an ESCALAB250Xi X-ray photoelectron spectroscopy (XPS) (Thermo Fisher Scientific Inc., Waltham, MA, USA. The excitation source of XPS was Al K alpha (*hυ* = 1486.6 eV), the pass energy was about 100 eV, and the binding energy of internal standard contaminated carbon was 284.8 eV.

In order to avoid the interference of the water peak brought by the traditional KBr pellet pressing, the Attenuated Total Reflection Fourier Transform Infrared (ATR-FTIR) test using a 670-IR spectrometer (Varian Co., Mashhad, California, USA) was directly applied to the PBO fiber samples. The spectral range was 4000–400 cm^−1^ and the resolution was 0.1 cm^−1^.

Deionized water droplets were applied to a contact angle test on the PBO fiber surface using an OCA20 optical contact angle measuring instrument (DataPhysics Instruments GmbH, Stuttgart, Baden-Württemberg, Germany) at 20 °C and a relative humidity of 40%. The volume of deionized water used for the test was 5 μL, and the average value of the measured values at five different positions on the PBO fiber surface was taken as the contact angle of the sample.

Thermal stability analysis of PBO/PI composites with different interfaces was carried out under a nitrogen atmosphere using a Pyris 1 (PerkinElmer Co, Waltham, MA, USA) thermo-gravimetric analyzer. The sample mass was approximately 10 mg, the temperature range was from room temperature to 900 °C, and the rate of controlled heating was 10 °C/min.

The tensile test was carried out on an INSTRON 3367 universal material testing machine (INSTRON Corporation, Boston, Massachusetts, USA) in accordance with the Chinese standard GB/T 1040.2–2006. The hardness test was carried out according to the Chinese standard GB/T 3398.2–2008 on the XHRD–150 electric plastic Rockwell hardness tester (Laizhou Huayin Testing Instrument Co., Ltd., Laizhou, Shangdong, China). The diameter of the steel ball indenter was 6.350 mm, and the scale used was L. All the values were averages of five measurements.

**Figure 3:**
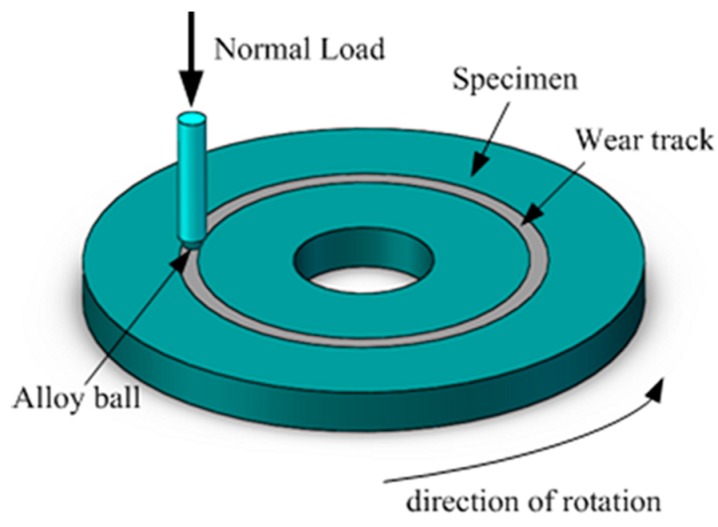
The schematic view of the working principle of the friction tester.

The tribological tests of PI composites were carried out on a High-Temperature Atmosphere Tribometer (QG–700, Lanzhou Zhongke Kaihua Technology Development Co., Ltd., Lanzhou, Gansu, China). Figure 3 is a schematic view of the working principle of the friction tester. The composite sample rotated with the chassis at a set speed, and the coupled alloy ball (89 HRA, 0.1 μm Ra) was fixed on the clamp. Before the test, the surface of the sample was polished with Al_2_O_3_ sandpaper, and then the surface of the friction specimen and the alloy ball was sufficiently washed with acetone. The tribological performance tests were carried out under the conditions of two representative temperatures (130 and 260 °C) for a test time of 60 min. The normal loads were 3, 6, 10, and 12 N, respectively, and the sliding speeds were 0.25, 0.5, 0.75, and 1 m/s, respectively. Three identical samples were performed under the same test conditions, and the average value was taken as the test result. The cross-sectional area of the wear scar was measured using a Nanofocus three-dimensional shape analyzer to calculate the abrasive volume V. The specific wear rate K of the composite was calculated by the formula:

(1) K=V/LF

where *V* denotes the abrasive volume; *F* denotes normal load; *L* denotes the total sliding distance.

The friction coefficient was automatically recorded by the friction tester, and the resulting data was the average of the last 30 min. The morphology of the relevant friction products was investigated with an environmental scanning electron microscope (ESEM, XL-30, Royal Philips, Amsterdam, The Netherlands).

## 3. Results and Discussion

### 3.1. Analysis of Surface Properties of PBO Fibers

**Figure 4:**
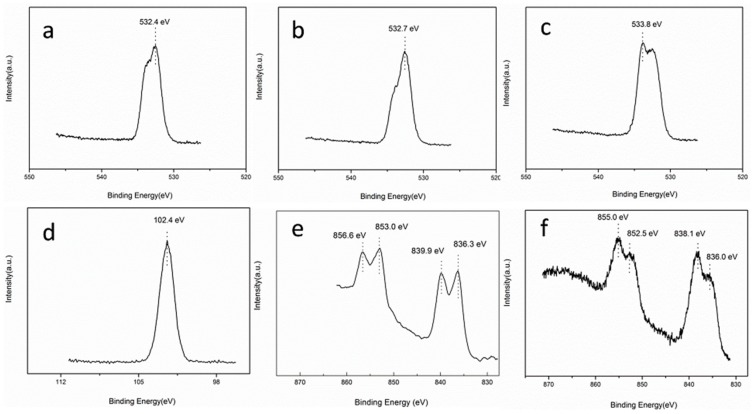
XPS narrow-spectra of (**a**) O_1s_ of PBO-Pre, (**b**) O_1s_ of PBO-APTES, (**c**) O_1s_ of PBO-La, (**d**) Si_2p_ of PBO-APTES, (**e**) La_3d_ of LaCl_3_, and (**f**) La_3d_ of PBO-La.

Figure 4a–f depicts the XPS narrow-spectra of the PBO-Pre, PBO-APTES, and PBO-La fibers. It can be seen from Figure 4a that the characteristic peak at the binding energy of 532.4 eV is assigned to the O_1s_ of the PBO-Pre fiber. After the APTES modification, the characteristic peak of O_1s_ moves to the binding energy of 532.7 eV, which is 0.3 eV higher than that of the PBO-Pre fiber, as shown in Figure 4b. Moreover, the presence of Si_2p_ is detected on the surface of the PBO-APTES fiber, and its peak appears at 102.4 eV (Figure 4d), which corresponds to the Si−O bond in the APTES. The APTES modification can introduce silanol groups on the surface of the fiber, which is probably the reason for the increase of the binding energy of O_1s_, indicating that the APTES modification forms an oxygen-containing functional group on the surface of the fiber. For the PBO-La fiber, the characteristic peak of O_1s_ appears at 533.8 eV, and the binding energy increases by 1.4 eV compared to the PBO-Pre fiber (Figure 4c). Meanwhile, the characteristic peaks of La3d can be observed at 836.0, 838.1, 852.5, and 855.0 eV, as shown in Figure 4f.

It can be discovered that the La_3d_ peaks are chemically shifted toward the low binding energy compared with the characteristic peaks of La_3d_ in LaCl_3_ (Figure 4e). These results may be interpreted as the fact that the coordination number of the lanthanum element is large. During performing the La salt modification, the lanthanum ion (La^3+^) is coordinately bonded to the oxygen element on the surface of the PBO fiber, and attracts the outer electrons of oxygen toward it [20]. Therefore, the binding energy of the La_3d_ peaks is decreased and the binding energy of the O_1s_ peak is increased.

**Figure 5:**
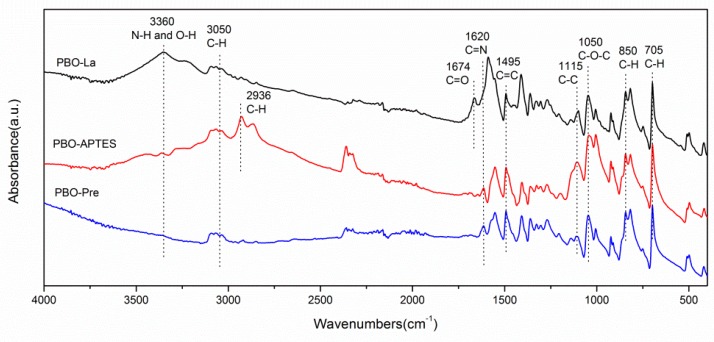
ATR-FTIR spectra of the PBO-Pre fiber, the PBO-APTES fiber, and the PBO-La fiber.

ATR-FTIR spectra of the PBO-Pre fiber, the PBO-APTES fiber, and the PBO-La fiber are shown in Figure 5. By comparison, it can be found that all three PBO fibers contain characteristic peaks corresponding to the molecular structure of PBO: The characteristic peaks at approximately 1620, 1050, and 3050 cm^−1^ are attributed to the C=N stretch, C–O–C stretch, and aromatic C–H stretch, respectively [21]. Peaks at 705 and 850 cm^−1^ can correspond to the plane flexural vibration peaks of C–H. Peaks at 1115 and 1495 cm^−1^ can be assigned to the skeletal vibration of C–C and the stretching vibration of C=C of the benzene ring [14]. For the PBO-APTES, a new characteristic peak appears near 2936 cm^−1^, which is corresponds to the stretching vibration of C–H groups in APTES. Furthermore, a small characteristic peak appears near 3360 cm^−1^, corresponding to O–H stretching vibration, which indicates that APTES modification generates a small amount of hydroxyl functional groups on the surface of PBO fiber [22]. The spectrum of PBO-La exhibits a broad characteristic peak at approximately 3360 cm^−1^, which is attributed to the combination of stretching vibrations of N–H and O–H [23]. In addition, an obvious characteristic peak at 1674 cm^−1^, attributed to carbonyl (C=O), occurs [24]. This demonstrates that a large amount of oxygen-containing reactive functional groups is attached to the PBO fiber surface after the modification of the La salt solution, which increases the activity of PBO fiber surface.

**Figure 6:**
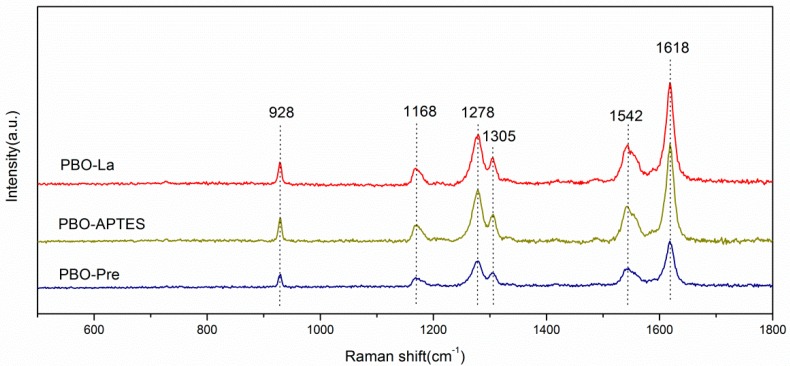
Raman spectra of PBO-Pre, PBO-APTES and PBO-La fibers.

The Raman spectra of the PBO-Pre, PBO-APTES, and PBO-La fibers are shown in Figure 6. The strong peak observed at 1618 cm^−1^, along with other characteristic peaks at 928, 1168, 1278, 1305, and 1542 cm^−1^, are assigned to the vibration mode of the backbone p-phenylene ring [25]. Compared with the PBO-Pre fiber, the PBO-APTES and PBO-La fibers do not exhibit distinct characteristic peaks, indicating that APTES and La salt modification have no appreciable influence on the molecular structure of PBO fibers.

**Figure 7:**
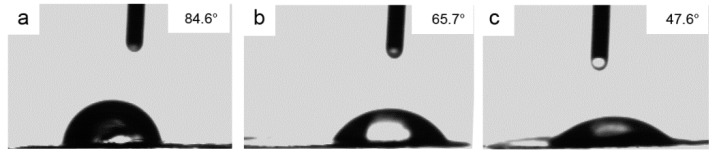
Contact angle (CA) images of deionized water droplets on the surface of the (**a**) PBO-Pre, (**b**) PBO-APTES, and (**c**) PBO-La fibers.

Figure 7 presents the result of contact angle (CA) measurements of the PBO-Pre, PBO-APTES, and PBO-La fibers, respectively. It can be seen from Figure 7a that the water drop adheres to the fiber surface in a beaded morphology, and the CA is about 86.1° ± 2.42°, indicating that the surface of the PBO-Pre fiber is poor in hydrophilicity and contains less polar groups. After surface modification, the CA is reduced. As shown in Figure 7b, the CA of the PBO-APTES fiber is about 66.3° ± 1.94°, which means that the hydrophilicity of the fiber after APTES modification is greatly improved, and the surface wetting property is improved. It can also be observed in Figure 7c that the CA of the PBO-La fiber is reduced to a minimum of 46.2° ± 1.19°. These results manifest that both of the APTES and La salt modifications introduce polar groups on the PBO fiber surface, which increases the chemical activity of the fiber surface, while La salt modification is a more effective approach to improve the surface reactivity of fiber surface than APTES modification.

### 3.2. TGA Study of PI Composites and the PBO-Pre Fiber

**Figure 8:**
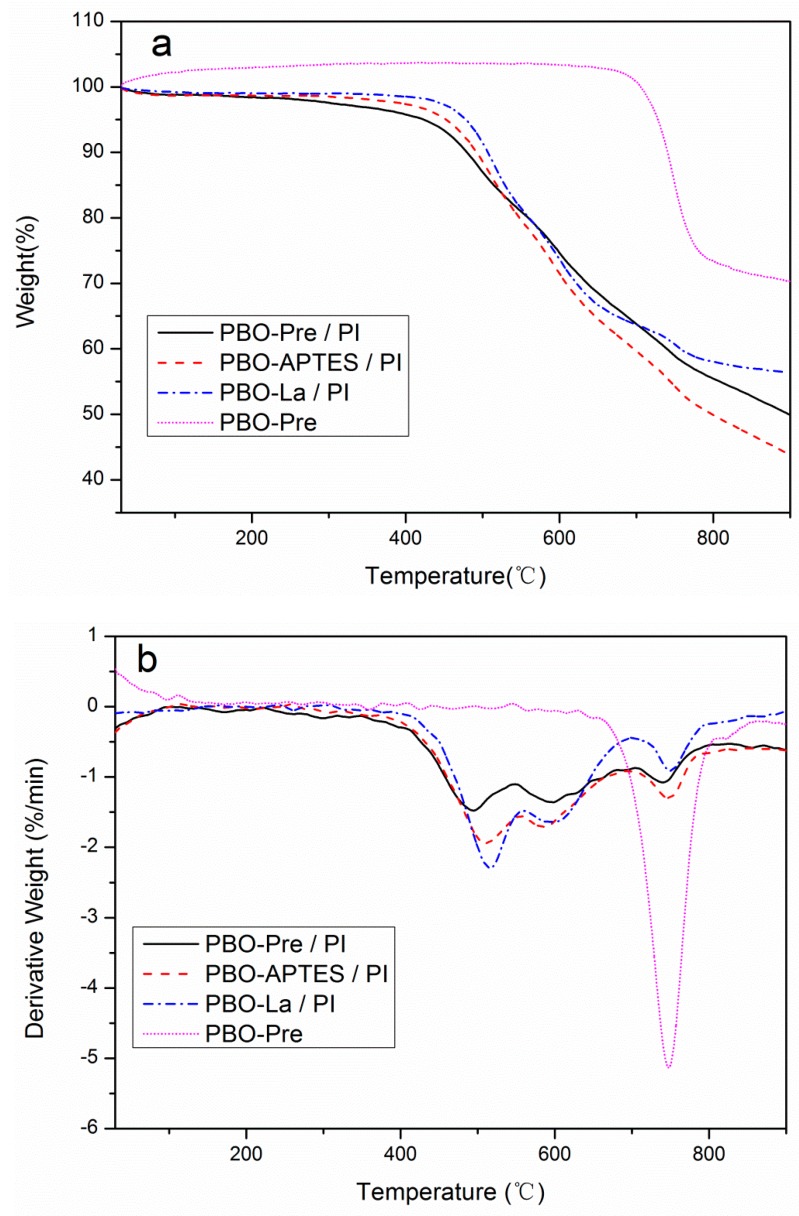
(**a**) TGA, (**b**) DTG of the PBO-Pre fiber and PI composites.

Figure 8a,b displays the thermogravimetric analysis (TGA) and differential thermogravimetric analysis (DTG) curves of the PBO-Pre fiber and PI composites, respectively. Table 1 lists the decomposition temperatures under different weight loss and the maximum rate of weight loss. As can be seen from Figure 8a, *T*_5%loss_ and *T*_10%loss_ of the PBO-APTES/PI and PBO-La/PI composites are improved obviously compared to those of the PBO-Pre/PI composite, and the PBO-La/PI composite is superior to PBO-APTES/PI. In addition, the PBO-Pre fiber has excellent thermal stability, and barely loses weight below 700 °C, which is similar to the result of the literature [26]. The initial thermal decomposition of various composites is primarily derived from the PI matrix. Therefore, the enhancing thermal stability by surface modification can be mainly ascribed to the fact that surface-modified PBO fibers can form a high interfacial adhesion with the PI matrix, which hinders the thermal motion of the molecular chain of the PI matrix and inhibits the process of volatile decomposed products out of the polymer matrix to become the gas phase. It can be seen from Figure 8b that the DTG curves of the three PI composites each contain a maximum “concave peak”, which indicate the temperatures corresponding to maxima of mass loss rate for various PI composites. The surface modification of the PBO fiber increases the temperature value corresponding to the peak in different extent. Compared with the PBO-Pre/PI composite, the *T*_max_ of the PBO-APTES/PI and PBO-La/PI composites is increased by 14 and 24 °C, respectively. These results further verify that surface modification can improve the thermal stability of PI composites, while La salt modification is a more effective approach to improve the thermal stability of PI composite than APTES modification. The possible reason is that the rare earth element is an inorganic element, and the product formed therefrom is more resistant to high temperatures than conventional organic modifiers.

### 3.3. Basic Mechanical Properties

**Figure 9:**
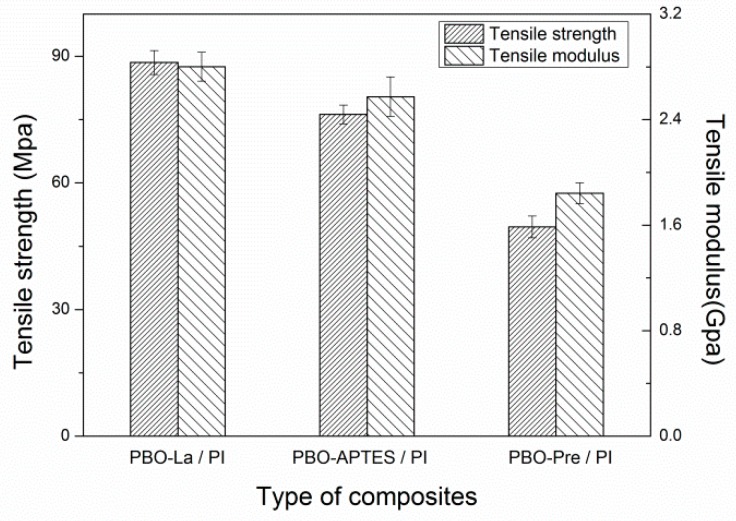
The tensile properties of the three composites at room temperature.

Figure 9 depicts the tensile properties of the three composites at room temperature. It can be seen that the tensile properties of the PBO-APTES/PI and PBO-La/PI composites are better than those of the PBO-Pre/PI composite. Compared with the PBO-Pre/PI composite, the tensile strength and elastic modulus of the composite modified by the APTES increased by about 53.8% and 39.8%, respectively, while the La salt modification increased the yield by about 78.6% and 52.1%. The test results show that while both modified methods can improve the interface bonding state of the composite, the La salt modification is more effective.

**Figure 10:**
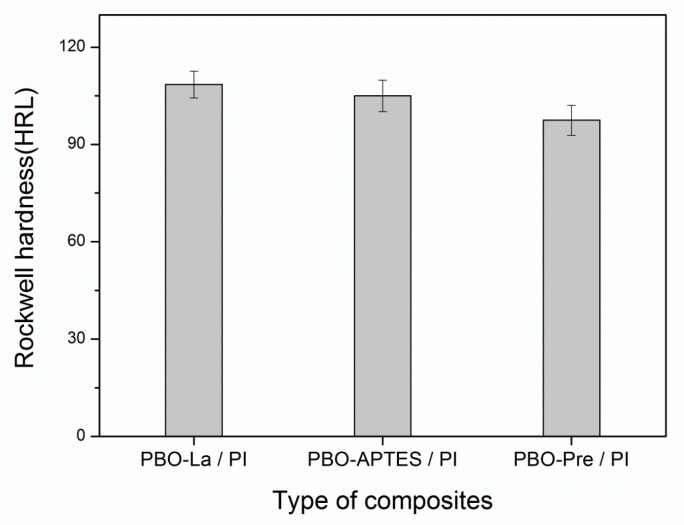
The hardness of the three composites at room temperature.

APTES modification and La salt modification have little effect on the hardness of the PI composite, as shown in Figure 10. This may be related to the fact that the PBO fiber as the reinforcing phase is an organic fiber and has a low hardness.

### 3.4. Friction and Wear Behavior

#### 3.4.1. Ambient Temperatures of 130 °C

**Figure 11:**
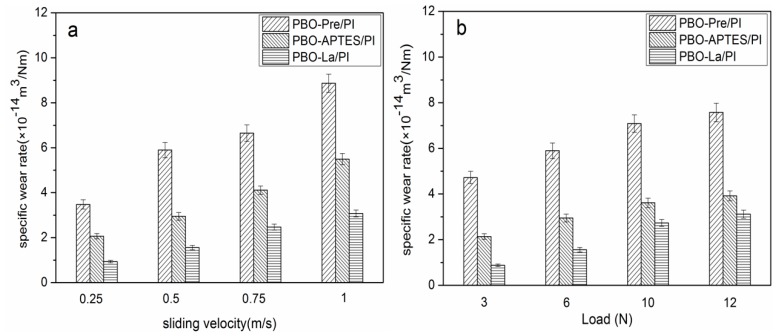
The specific wear rate of PI composites in a 130 °C environment as a function of (**a**) sliding velocity (load: 6 N) and (**b**) load (velocity: 0.5 m/s).

The specific wear rate of the PBO-Pre/PI, PBO-APTES/PI, and PBO-La/PI composites in a 130 °C environment as a function of sliding velocity (load: 6 N) and load (velocity: 0.5 m/s) are shown in Figure 11a,b, respectively. It can be seen that the specific wear rates of the three composites increase with the increase of the sliding velocity and the normal load. This is probably because the increase in the sliding velocity causes the friction surface of the composite to generate more heat, and the increase in the load exacerbates the plastic deformation of the friction surface. Both of these factors aggravate the adhesive transfer of the resin matrix to the surface of the counterpart. Under the same sliding velocity and load, the surface modifications significantly improve the wear resistance of the PI composites, while La salt modification is superior to APTES modification.

**Figure 12:**
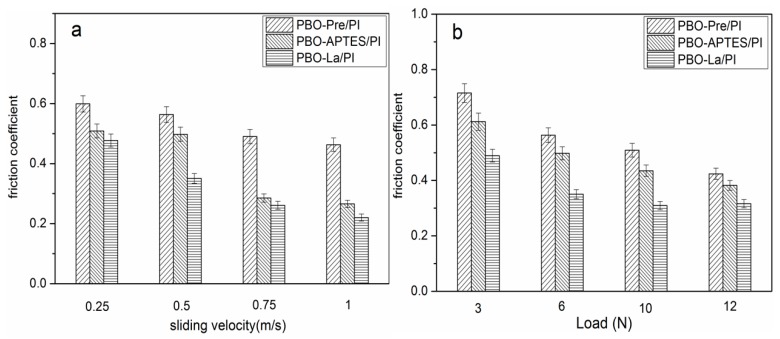
The friction coefficient of PI composites in a 130 °C environment as a function of (**a**) sliding velocity (load: 6 N) and (**b**) load (velocity: 0.5 m/s).

Figure 12a,b reveals the relationship of the friction coefficient of the three composites with sliding velocity (load: 6 N) and load (velocity: 0.5 m/s), respectively. The friction coefficient generally decreases with the increase of sliding velocity and load. The increase of the sliding velocity increases the temperature of the friction surface, causing the surface of the composite matrix to soften, which is beneficial to reduce the frictional resistance. The increase of the load promotes the transfer of the PI resin to the surface of the counterpart, which constitutes the friction between the PI–PI, and reduces the direct friction between the composite and the counterpart, thereby causing the friction coefficient of the composite to decrease. During the friction process, the PBO fiber as the reinforcing phase bears the main load between the composite and the surface of the counterpart, and becomes the object of preferential wear, reducing the actual contact area of the composite matrix with the counterpart and the shear-tear-strength of the bond point. Under the same sliding velocity and load, the friction coefficient of the PBO-La/PI composite is the smallest, the friction coefficient of the PBO-Pre/PI composite is the largest, and the friction coefficient of the PBO-APTES/PI composite is between them.

**Figure 13:**
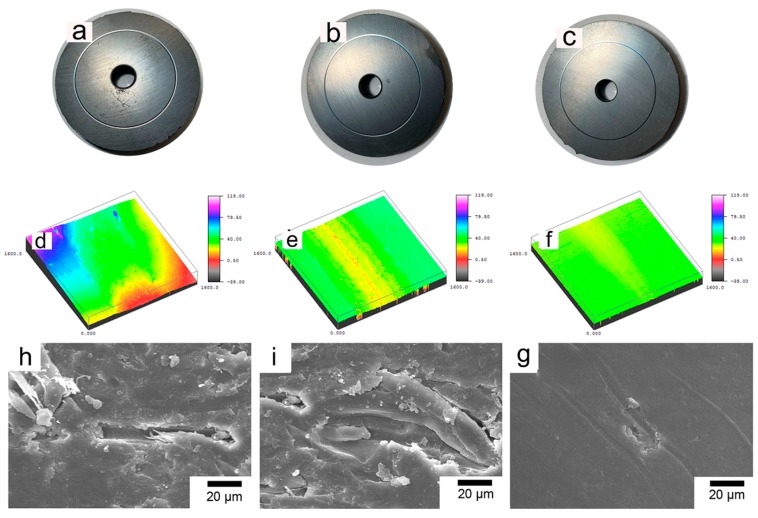
The appearance and SEM photographs of the wear scars of the (**a**,**d**,**h**) PBO-Pre/PI, (**b**,**e**,**i**) PBO-APTES/PI, and (**c**,**f**,**g**) PBO-La/PI composites.

Figure 13 exhibits the general appearance, three-dimensional profiles, and SEM photographs of the wear tracks of the three composites. The test parameters are the sliding velocity of 0.5 m/s and the load of 6 N. Figure 13a–c shows the general appearance of test samples after the friction test. It is intuitive that the wear scar of the PBO-Pre/PI composite is much wider and deeper than that of the APTES/PI and PBO-La/PI composites, and the wear scar of the PBO-La/PI composite is the slightest, as shown in Figure 13d–f. The wear of the PBO-Pre/PI composite is severe and the friction surface is rough. The interfacial adhesion property of the composite is poor, and the stress cannot be effectively transferred from the PI matrix to the PBO fiber, resulting in a decrease in the bearing capacity of the PI matrix around the PBO fiber. The friction surface is mainly characterized by plastic deformation and adhesive wear, as shown in Figure 13h. After the APTES modification, the wear of the composite is improved, and the phenomenon that the PBO fiber is pulled out from the matrix is alleviated. However, there are still obvious pores between the PBO fiber and the PI matrix, as shown in Figure 13i, and the pits in the PI matrix induce more stress concentration points, which in turn increases the wear of the composite material. It can be seen from Figure 13g that the wear surface of the PBO-La/PI composite is relatively smooth, the PBO fiber is tightly combined with PI resin, and the interface crack is small, forming a good interface bond, which can effectively support the load from the counterpart. The wear mechanism is mainly characterized by slight abrasive wear and adhesive wear.

**Figure 14:**
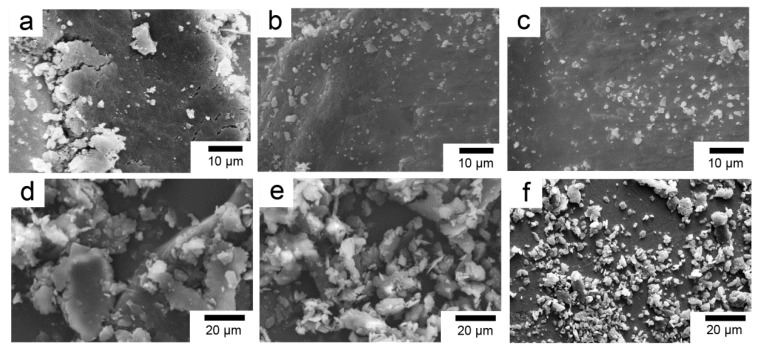
SEM photographs of the counterface and wear debris corresponding to the (**a**,**d**) PBO-Pre/PI, (**b**,**e**) PBO-APTES/PI, and (**c**,**f**) PBO-La/PI composites.

In order to study the friction and wear behavior of the three composites more comprehensively, the morphologies of the counterface and wear debris were analyzed by SEM, and the results are shown in Figure 14. The test parameters are the sliding velocity of 0.5 m/s and the load of 6 N. The transfer film formed on the counterface rubbed with the PBO-Pre/PI composite has a large thickness, a low compact degree, and many fine cracks (Figure 14a), which is probably due to the fact that the PBO fibers are easily peeled off from the friction surface, and the PI matrix is largely transferred to the counterface. It can be further found from the morphology of the wear debris of the PBO-Pre/PI composite (Figure 14d) that the bonding force between the transfer film and the counterface is weak. Under the action of frictional heat and load, the transfer film and the composite have an adhesive action and are broken into large-sized and thick sheet-like wear debris to peel off, so that the specific wear rate of the PBO-Pre/PI composite is large. The compact degree of the transfer film formed on the counterface rubbed with the PBO-APTES/PI composite is improved, but some of the transfer film is still relatively thick with a slight sticking phenomenon (Figure 14b). The resulting wear debris is medium-sized, with a small amount of PBO fiber intermixed (Figure 14e). This indicates that the interfacial adhesion between the PBO fiber and the PI matrix is improved, thereby inhibiting the excessive transfer of the PI matrix and the formation of large flakes. The transfer film formed on the counterface rubbed with the PBO-La/PI composite has good integrity, uniformity, continuity, and compactness, and only micro-cutting marks and slight sticking phenomenon occur (Figure 14c), which indicates that the La salt modification can enhance the bonding force between the transfer film and the counterface. The corresponding wear debris is mostly in the form of fine particles (Figure 14f). This result is most likely since the PBO fiber undertakes the main load between the composite and the counterface during the friction process and becomes the object of preferential wear. Thereby, the probability of the PI matrix being sheared is reduced, the peeling of the large sheet is suppressed, and the wear resistance of the composite is greatly enhanced.

#### 3.4.2. Ambient temperatures of 260 °C

**Figure 15:**
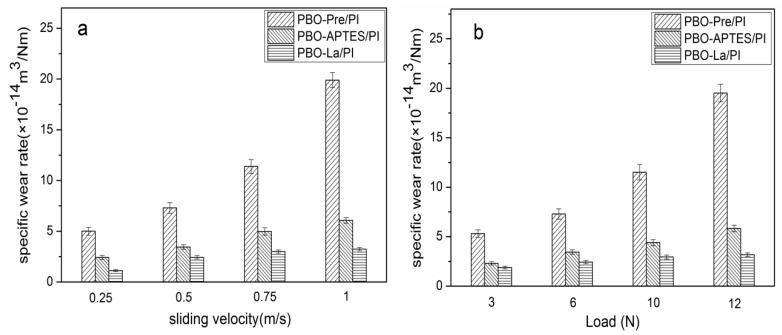
The specific wear rate of PI composites in a 260 °C environment as a function of (**a**) sliding velocity (load: 6 N) and (**b**) load (velocity: 0.5 m/s).

Figure 15 depicts the specific wear rate of three composites in the 260 °C environment as a function of sliding velocity and load. It can be seen that the relation between the specific wear rate and the sliding velocity and the load is similar to that in the 130 °C environment, except that the value of the specific wear rate is increased under the same test parameters. The test parameters have a great influence on the PBO-Pre/PI composite, especially in the case of high sliding velocity and heavy load, while the specific wear rate of the PBO-La/PI composite is least affected by the test parameters.

**Figure 16:**
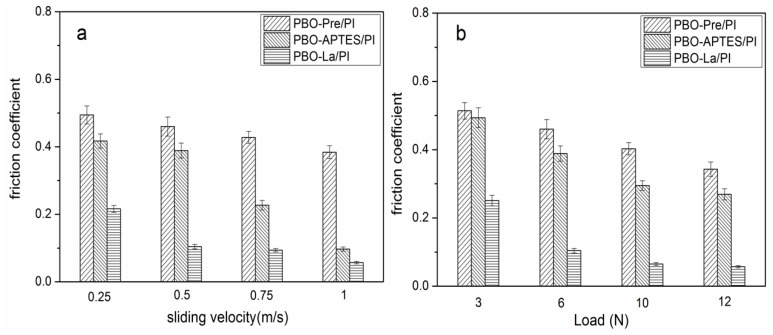
The friction coefficient of PI composites in a 260 °C environment as a function of (**a**) sliding velocity (load: 6 N) and (**b**) load (velocity: 0.5 m/s).

Figure 16 shows the relationship of the friction coefficient of the three composites in the 260 °C environment as a function of sliding velocity and load. Compared with the test results in the 130 °C environment, it can be seen that the corresponding friction coefficients are reduced. The interfacial bonding performance of the composite determines whether the PBO fiber can bear the main load between the friction pair, and whether the PBO fiber can fully exert its excellent lubricating performance, thus playing a key role in the antifriction performance of the composite. Under the same test parameters, the La salt-modified PBO/PI composite has the lowest friction coefficient.

**Figure 17:**
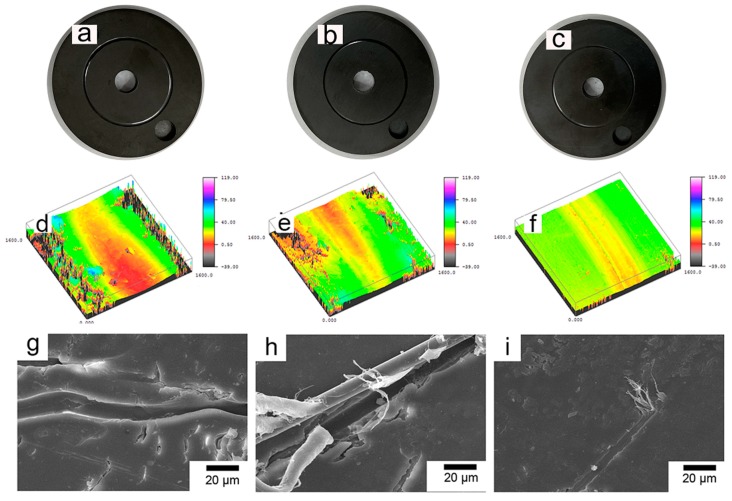
The appearance and SEM photographs of the wear scars of the (**a**,**d**,**g**) PBO-Pre/PI, (**b**,**e**,**h**) PBO-APTES/PI, and (**c**,**f**,**i**) PBO-La/PI composites.

The general appearance, three-dimensional profiles, and SEM photographs of the wear scars of the three composites in the 260 °C environment are exhibited in Figure 17. The test parameters are the sliding velocity of 0.5 m/s and the load of 6 N. Figure 17a–c are the general appearance of test samples after the friction test. The wear scar width and depth of the corresponding composites increase to varying degrees compared to the wear scar morphology in the 130 °C environment, as shown in Figure 17d–f. The wear surface of the PBO-Pre/PI composite exhibits a large number of fatigue cracks and grooves left by the fibers peeling off the PI matrix, accompanied by plastic deformation and adhesive wear characteristics, as shown in Figure 17g. There is no obvious fatigue crack on the wear surface of the PBO-APTES/PI composite, but the PBO fiber still gradually peels off from the PI matrix, as shown in Figure 17h. The PBO-La/PI composite has the slightest wear degree and smooth wear surface. The PBO fiber and PI matrix form a good interface. During the friction process, the PBO fiber continuously withstands friction and causes “peeling” on the surface, as shown in Figure 17i.

**Figure 18:**
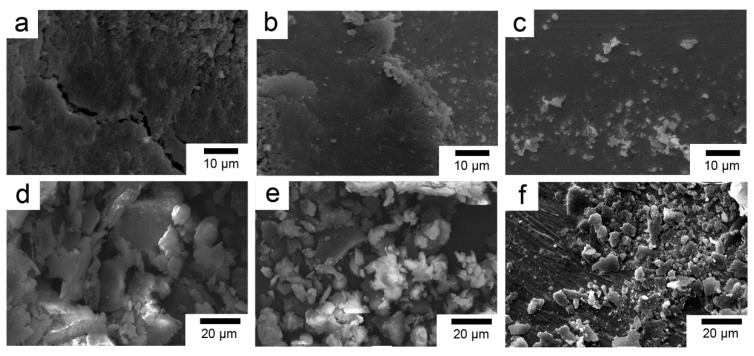
SEM photographs of the counterface and wear debris corresponding to the (**a**,**d**) PBO-Pre/PI, (**b**,**e**) PBO-APTES/PI, and (**c**,**f**) PBO-La/PI composites.

Figure 18 presents the SEM photographs of the counterface and wear debris corresponding to the three composites. The test parameters are the sliding velocity of 0.5 m/s and the load of 6 N. The transfer film formed on the counterface rubbed with the PBO-Pre/PI composite is thick and loose. The transfer film produces large cracks under the action of high friction heat and normal load, and further breaks into wear debris and peels off (Figure 18a). There is also a part of the wear debris formed by the friction of the counterpart due to the loss of support of the PBO fiber. The wear debris is dominated by large and thick blocks (Figure 18d), and the composite has poor wear resistance. The density of the transfer film formed on the counterface rubbed with the PBO-APTES/PI composite is improved, but the thickness of the transfer film formed is not uniform, accompanied by some fine cracks (Figure 18b). The amount of large block wear debris is reduced, while the number of medium block wear debris is increased (Figure 18e). The transfer film is relatively uniform, dense, and thin, with only micro-cutting traces and slight sticking (Figure 18c). The PBO-La/PI composite has a tough interface at high temperature, which inhibits large peeling of the PI matrix. The formed wear debris appears as a small block (Figure 18f), which reduces the fatigue wear and adhesive wear of the composite.

## 4. Conclusions

In summary, the friction and wear behaviors of PI composites with different interface properties were investigated at 130 °C and 260 °C, respectively. The results show that the friction coefficient of the three composites decreases with the increase of sliding velocity and load at two temperatures, and the specific wear rate increases. The test parameters have a great influence on the PBO-Pre/PI composite, especially in the case of high sliding velocity and heavy load, while the specific wear rate of the PBO-APTES/PI and PBO-La/PI composites are less affected. Under the same sliding velocity and load, the surface modifications significantly improve the properties of antifriction and wear resistance of the PI composites, while La salt modification is superior to APTES modification. Some oxygen-containing reactive functional groups are attached to the PBO fiber surface after the modification of APTES and La salt, thereby improving the interfacial adhesion properties of the PBO fiber and the PI matrix. Thermogravimetric analysis indicates that surface modification can increase the performance of high temperature resistance of PI composites, while La salt modification is a more effective approach to improve the thermal stability of PI composite than APTES modification.

## Figures and Tables

**Table 1:** Characteristic decomposition temperatures of the PBO-Pre fiber and PI composites at different mass losses.

Material Samples	*T*_5%loss_ (°C)	*T*_10%loss_ (°C)	*T*_max_ (°C)
PBO-Pre/PI	423	479	493
PBO-APTES/PI	452	492	507
PBO-La/PI	478	506	517
PBO-Pre	728	740	747
